# Haploinsufficiency of *Col5a1* causes intrinsic lung and respiratory changes in a mouse model of classical Ehlers‐Danlos syndrome

**DOI:** 10.14814/phy2.15275

**Published:** 2022-04-19

**Authors:** Jordan Fett, Milena Dimori, John L. Carroll, Roy Morello

**Affiliations:** ^1^ 12215 Department of Pediatrics University of Arkansas for Medical Sciences Little Rock Arkansas USA; ^2^ 12215 Department of Physiology & Cell Biology University of Arkansas for Medical Sciences Little Rock Arkansas USA; ^3^ 12215 Department of Orthopaedic Surgery University of Arkansas for Medical Sciences Little Rock Arkansas USA; ^4^ 12215 Division of Genetics University of Arkansas for Medical Sciences Little Rock Arkansas USA

**Keywords:** *Col5a1*, collagen, Ehlers‐Danlos, pediatrics, respiratory mechanics

## Abstract

The Ehlers‐Danlos syndromes (EDS) are inherited connective tissue diseases with primary manifestations that affect the skin and the musculoskeletal system. However, the effects of EDS on the respiratory system are not well understood and are described in the literature as sporadic case reports. We performed histological, histomorphometric, and the first in‐depth characterization of respiratory system function in a mouse model of classical EDS (cEDS) with haploinsufficiency of type V collagen (*Col5a1*+/−). In young adult male and female mice, lung histology showed reduced alveolar density, reminiscent of emphysematous‐like changes. Respiratory mechanics showed a consistent increase in respiratory system compliance accompanied by increased lung volumes in *Col5a1*+/− compared to control mice. Flow–volume curves, generated to mimic human spirometry measurements, demonstrated larger volumes throughout the expiratory limb of the flow volume curves in *Col5a1*+/− compared to controls. Some parameters showed a sexual dimorphism with significant changes in male but not female mice. Our study identified a clear respiratory phenotype in the *Col5a1*+/− mouse model of EDS and indicated that intrinsic respiratory and lung changes may exist in cEDS patients. Their potential impact on the respiratory function during lung infections, other respiratory disease processes, or insults may be significant and justify further clinical evaluation.

## Graphical abstract

We used mice that are haploinsufficient for Col5a1 (Col5a1+/−), a well‐accepted model of classical EDS type, and demonstrated the presence of primary changes in the lung parenchyma that are reminiscent of emphysema. We then performed a detailed analysis of the respiratory mechanics, lung volumes, and flow–volume curves and show that both male and female animals are significantly different from controls, including elevated respiratory system compliance and other changes that are consistent with a respiratory system that is more readily distended to larger volumes.

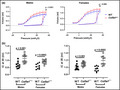

## 1 INTRODUCTION

The Ehlers‐Danlos syndromes (EDS) represent a genetically and clinically heterogeneous group of inherited connective tissue diseases (for a review see Malfait et al., 2020) that share clinical features including joint hypermobility, tissue fragility, and skin changes with a spectrum of severity that goes from subclinical to life‐threatening. The 2017 international Ehlers‐Danlos syndrome classification describes 13 different types of EDS and a fourteenth rare subtype has recently been identified (Blackburn et al., 2018; Malfait et al., 2017). Variants in 20 distinct genes can cause EDS and negatively affect the extracellular matrix (ECM). Primary defects in fibrillar collagens (types V, III and I), in their processing, or in the synthesis of proteoglycans are associated with the majority of EDS cases (Malfait et al., 2020). Different EDS types have a characteristic profile of clinical features and major criteria for each type have been described (Malfait et al., 2020). These can be helpful in the differential diagnosis, which is often challenging. Although the disease prevalence for rare forms of EDS has not been determined, the incidence of some of the most common types of EDS, such as classical EDS (cEDS) and vascular EDS (vEDS) is estimated at approximately 1 in 20,000 and 1 in 50,000–200,000, respectively (Byers et al., 2017; Ghali et al., 2019; Symoens et al., 2012).

Although EDS is frequently considered a disorder of the integumentary, vascular and musculoskeletal systems, most EDS types are systemic conditions and can affect additional organs and systems of the body, including the gastrointestinal, genitourinary, cardiovascular, respiratory, craniofacial, and ocular systems (Malfait et al., 2020). The effects of EDS on the respiratory system are not well characterized and are mostly published as case reports. EDS has been associated with the development of respiratory complications including bullous emphysema (Ruggeri et al., 2015), pulmonary cysts and nodules (Berezowska et al., 2018; Herman & McAlister, 1994; Kawabata et al., 2010), pneumothorax (Boone et al., 2019; Dowton et al., 1996; Kadota et al., 2016; Malfait et al., 2020; Nakagawa et al., 2015; Shalhub et al., 2019), hemoptysis (Dowton et al., 1996; Hatake et al., 2013), tracheobronchomegaly (Girit et al., 2018), asthma symptoms, and other respiratory problems such as obstructive sleep apnea (Gaisl et al., 2017; Garcia Saez et al., 2014; Harris et al., 2013; Morgan et al., 2007; Stoberl et al., 2019). In one case, series of 252 patients with EDS, including 33 with cEDS, clinically significant pulmonary symptoms were described in nearly half of all patients (Sheehan et al., 2017). In the cEDS cohort, clinically significant shortness of breath and chest pain was found in a third of the patients (Sheehan et al., 2017).

The respiratory manifestations in EDS have recently been reviewed (Chohan et al., 2021; Parducci, 2021) and were described in patients affected with vEDS, cEDS and hEDS (hypermobile) types. Respiratory abnormalities may be associated with significant morbidity and may be under‐recognized in EDS (Chohan et al., 2021; Parducci, 2021). Importantly, although connective tissue laxity is a prominent feature of EDS, its effects at the level of the lung tissue and on the respiratory function of patients with EDS have not been described. As studies in humans are difficult to perform and often underpowered in rare diseases, the availability of a validated mouse model that mimics the condition in humans is an important tool for a more robust and adequately powered research approach. The availability of a validated mouse model of classical EDS allows for correlations between respiratory system function, lung structure, and a specific collagen‐related mutation, which is not possible in studies of human subjects. Therefore, we used a well‐accepted mouse model for cEDS (*Col5a1*+/−) (Wenstrup et al., 2004, 2006) to study the effects of *Col5a1* haploinsufficiency on the lung parenchyma and perform accurate measurements of respiratory system mechanics and pulmonary function.

## 2 METHODS

### 2.1 Mice

*Col5a1*+/− mice were kindly provided by Dr. David Birk (University of South Florida, Tampa, FL) and their generation was described elsewhere (Wenstrup et al., 2006). The *Col5a1* mice were maintained on a C57/BL6 pure background and were genotype by PCR protocol using the following primers: *Col5a1*_FORWARD 5'‐ CTGTAGAGGTTTGATCTTAGGGCG‐3' and REVERSE‐1 5'‐CATCATAAACCATCTACTATCGGG‐3' and REVERSE‐2 5'‐CTTCTATCGCCTTCTTGACGAGTT‐3'. The product of the reaction is a 500 base pair (bp) for the wild‐type (WT) and 700bp for heterozygotes (*Col5a1*+/−). Mice were housed in a pathogen‐free facility, with unlimited access to water and standard rodent chow and an environmental 12‐hour light/dark cycle. All animal studies were performed under UAMS IACUC approved protocol (AUP # 3845) and in accordance with local, state, and U.S. Federal regulations.

### 2.2 FlexiVent respiratory measurements

Both male and female *Col5a1*+/− mice (*n* = 10 and *n* = 11, respectively) and their WT littermate controls (*n* = 10 and *n* = 9, respectively) were studied between 3–4 months of age. Mice were anesthetized with a mixture of Ketamine (100 mg/kg I.P.) and Xylazine Hydrochloride (10 mg/kg I.P.), underwent surgical tracheostomy (as previously described in Dimori et al., 2020) and were intubated with a beveled metal cannula (18 gauge, 0.5" long). The cannula was tied to the trachea with a silk suture #3 and then connected to a Flexivent small animal ventilator (Scientific Respiratory Equipment (SCIREQ), Montreal, Quebec, Canada) which was configured with an F2 module without nebulizer and operated by FlexiWare software v.5.1. Animals were ventilated with a respiratory rate of 150 breaths/min, a tidal volume 10 mL/kg, and a PEEP 3 cm H_2_O. After assessment of adequate ventilation, the mice were paralyzed with succinylcholine (7.5 mg/kg I.P.) followed by 3 minutes of ventilation to allow the drug to reach its effect and stabilize the subject. The ventilator was programmed to perform the following sequence of perturbations: Deep inflation (6s at 30 cm H_2_O), Snap shot‐150 (150 breath/min 2.5 Hz), Quick prime‐3 (3 s, 1–20.5 Hz), NPFE (negative pressure‐driven forced expiration to emulate spirometry), and Partial pressure–volume (PV) curve. This set of the maneuvers was repeated 3 time with one‐minute interval between each set, and at the end, a terminal procedure to record the full pressure–volume curve was performed. All experiments were performed in a closed chest wall configuration.

#### 2.2.1 Deep inflation

opening pressure was increased to 30 cm H_2_O over 3 s before being held for three additional seconds. This maneuver is repeated multiple times to allow for standardization of volumes as well as recruitment of alveoli. The deep inflation maneuver was utilized to approximate inspiratory capacity.

#### 2.2.2 Forced oscillation techniques

respiratory mechanics were evaluated utilizing 1.2 s, 2.5 Hz single‐frequency forced oscillation maneuver (Snapshot 150 perturbation). Applying a single compartment model to output data yields measurements of respiratory system resistance (R_rs_), elastance (E_rs_), and compliance (C_rs_). Next, a broadband low‐frequency‐forced oscillation maneuver via volume‐driven perturbation (Quick prime‐3) delivered volumes over input frequencies ranging between 1 and 20.5 Hz. Using a constant phase model, the measured data are derived to calculate Newtonian resistance (R_n_), tissue damping (G), elastance (H), and hysteresivity (G/H or Eta).

#### 2.2.3 Pressure–volume curves

partial range pressure–volume curves were obtained on each mouse. Beginning at FRC (matched to PEEP), pressure was delivered to the lung at 5 cm H_2_O steps over 1 second intervals to a total pressure of 30 cm H_2_O until deflating the lung in a similar fashion back to starting FRC. Both pressure and volume were measured with each incremental plateau, which were then plotted as pressure–volume (PV) curves. The Salazar–Knowles experimental function (Salazar & Knowles, 1964) was fit to the deflation limb of the PV curves to determine parameters including quasi‐static compliance (C_st_), an estimate of inspiratory capacity (A), the shape constant showing the curvature of the upper deflation (K), and the area enclosed by the pressure–volume loop (Area), which correlates with airspace atelectasis.

#### 2.2.4 Negative pressure‐forced expiration maneuvers

A Negative pressure‐forced expiration (NPFE) maneuver was utilized to emulate spirometry data. First, the subject's lungs were inflated to 30 cm H_2_O (Total Lung Capacity) before exposure to vacuum output circuit resulting in rapid deflation of the lungs. This perturbation then plotted flow–volume curves and resultant forced expiratory volume at 0.1 seconds (FEV_0.1_), forced vital capacity (FVC) and the ratio between the two (FEV0.1/FVC). FEV _0.1_ is utilized in mouse models as analogous to FEV_1_ in human spirometry (Bonnardel et al., 2019).

#### 2.2.5 Full range pressure–volume curves and lung volume measurements

To obtain full range pressure–volume curves, TLC, RV and FRC, an automated series of perturbations was performed as described in Robichaud et al (Robichaud et al., 2017). In brief, recruitment maneuvers, followed by a partial pressure–volume loop to a pressure of 35 cm H_2_O were performed. Subjects were then ventilated with 100% oxygen for 5 min to flush nitrogen from the lungs. Following this, ventilation was stopped and the valves of the FlexiVent were closed. At this time, the pure oxygen within the lungs of the subject was absorbed, resulting in complete degassing of the lungs. This is a terminal procedure. The lungs were then inflated with a full‐range quasi‐static, ramp style, pressure–volume loop; whereby the lungs were inflated to a pressure of 35 cm H_2_O before being deflated to a pressure of −10 cm H_2_O. The TLC minus the IC, measured at 35 cm H_2_O, was used to calculate the FRC. The volume at −10 cm H_2_O, at the end of the full‐range PV loop, was used to characterize the RV.

### 2.3 Lung histology and morphometry

Lungs were harvested from a subset of mice (6 from each genotype and sex) following the Flexivent measurements. Mice were detached from the ventilator, a cervical dislocation was performed, and the cannula was then attached to a reservoir containing 10% buffered formalin. Lungs were fixed in situ at 25 cm H_2_O for 30 min, excised from the thoracic cavity and then fixed overnight in 10% buffered formalin. The next day, the fixed lung volume was measured (three measurements for each lung were taken and then averaged) utilizing Archimedes’ principle of water displacement (Limjunyawong et al., 2015). The lungs were then sequentially dehydrated in a series of increased concentration of ethanol and then embedded in paraffin. 5‐micron sections were obtained and stained with H&E for morphological analysis. Images from ten histological field were captured at 20× magnification using a Nikon Microscope (Eclipse E400) per each mouse. The ImageJ software plug‐in grid analysis was utilized to overlay a grid over each image of size equal to 745.28 × 558.96 μm with 8 horizontal lines and 10 vertical lines. The ImageJ Software was utilized to count the intersection of each alveolar wall with the grid line, allowing for a linear intercept distance for each image to be recorded. The mean linear intercept was calculated using the following equation Lm = horizontally (N) × (L) + vertically (N) × (L)/m where N is the number of time the transverse was placed on the tissue, L = length of the transverses, and m the sum of all the intercepts from each field. The internal lung surface area was also calculated using the formula ILSA = 4 × (lung fix volume)/MLI.

### 2.4 Statistical analysis

All measured parameters are presented as mean ± standard deviation and were analyzed with the Student's *t*‐test using two‐tailed distribution and two‐sample equal variance as appropriate. *P* < 0.05 were considered statistically significant and reported as such.

## 3 RESULTS

### 3.1 Lung histology, mean linear intercept, and alveolar surface area

**FIGURE 1:**
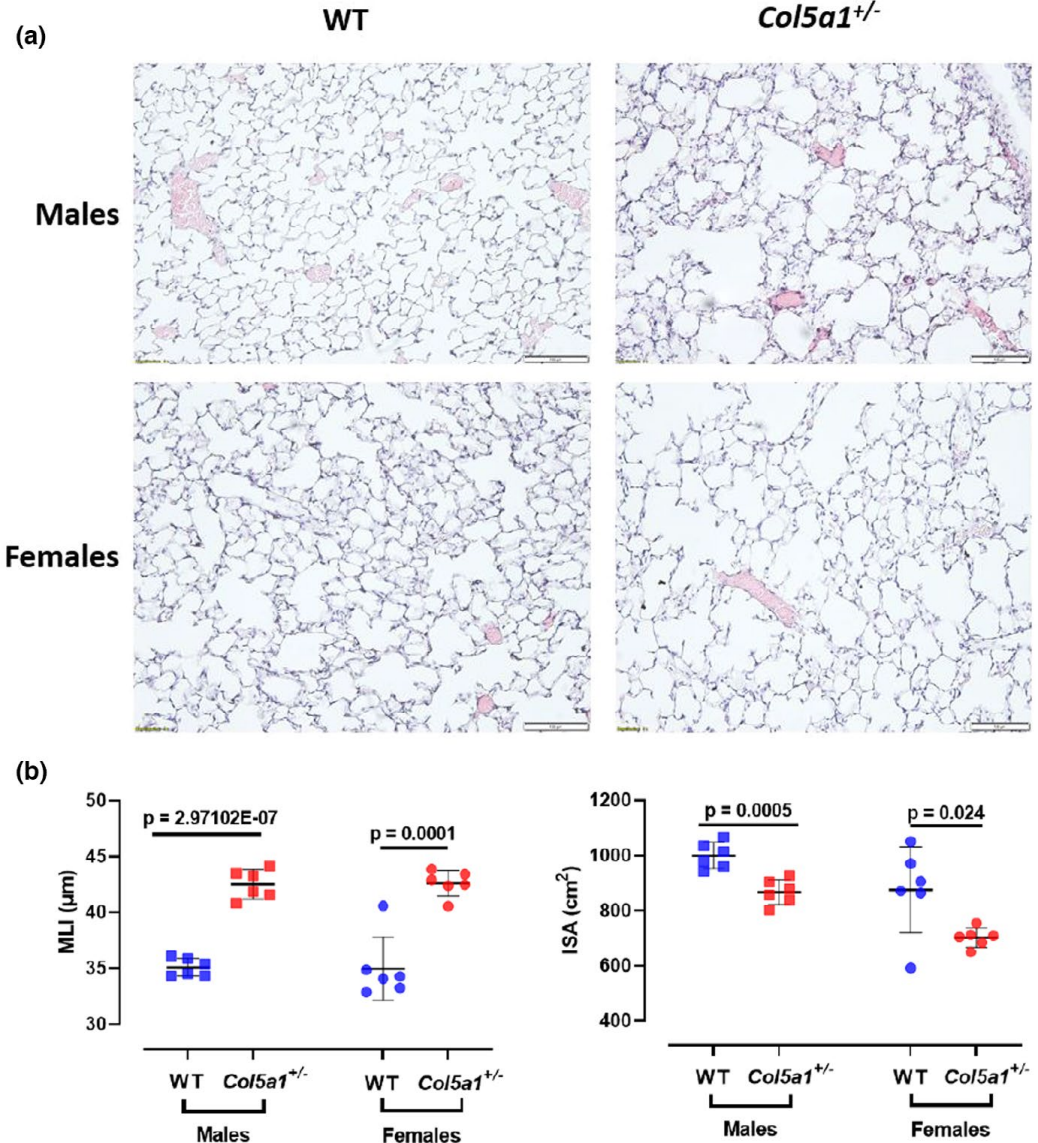
(a) Representative histological sections of WT and *Col5a1*+/− lung from 3–4 month‐old male and female mice. (b) Lung histomorphomeric measurements to quantify the lung parenchyma defect using the mean linear intercept (MLI) method and the calculation of the internal lung surface area (ISA) (*n* = 6/sex/genotype). Student's *t*‐test

Classical EDS is most often caused by heterozygous pathogenic variants in *COL5A1*, leading to haploinsufficiency and a quantitative reduction in α1(V), the alpha 1 chain of type V collagen (Ritelli et al., 2013; Schwarze et al., 2000; Symoens et al., 2012; Wenstrup et al., 2000). A mouse model with a *Col5a1* null allele (*Col5a1*+/−) was generated by others and shown to reproduce multiple features of cEDS (Wenstrup et al., 2006). To determine whether *Col5a1*+/− mice have intrinsic changes in their lung tissue compared to littermate controls, lung histology and histomorphometry were performed. Male and female mice were analyzed between 3 and 4 months of age (12–16 weeks). Their body weights were measured and no differences between males or females of the two genotypes were noted. Histological lung sections showed significant alterations in the alveolar structure of *Col5a1*+/− mice compared to WT controls. In males and females (*n* = 6/sex/genotype), *Col5a1*+/− lungs showed markedly enlarged alveolar spaces with a reduction in alveolar septal density (Figure 1a). These differences were quantified via measurement of the mean linear intercept (MLI) which was greater in both *Col5a1*+/− male and female (*n* = 6, *p* < 0.0001 and *p* = 0.001, respectively) compared to control mice (Figure 1b). The lung internal surface area (ISA), calculated from the MLI data, was markedly reduced in *Col5a1*+/− males (*p* = 0.0005) and females (*p* = 0.024), as a result of the overall loss in alveolar density (Figure 1b).

### 3.2 Respiratory system mechanics

#### 3.2.1 Forced oscillation technique

**FIGURE 2:**
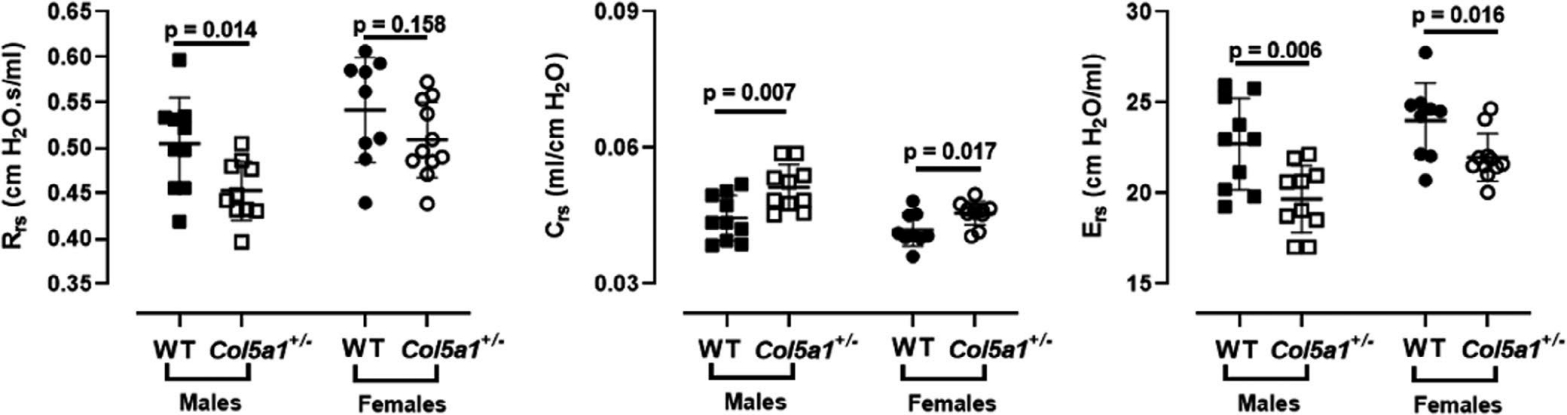
Measurements of the respiratory system resistance (R_rs_), compliance (C_rs_), and elastance (E_rs_) derived from the Snapshot 150 maneuver using the forced oscillation technique. (*n* = 10 males, *n* = 9–11 females). Student's *t*‐test

To evaluate if the observed lung morphological changes impact respiratory function, the forced oscillation technique (FOT) was used to detect possible differences in respiratory system mechanics between *Col5a1*+/− and WT mice. Respiratory system resistance (R_rs_), compliance (C_rs_), and elastance (E_rs_) were measured via single‐frequency forced oscillation (Snapshot 150). R_rs_ was significantly reduced in *Col5a1*+/− males compared to WT mice, but not in females (*n* = 10, *p* = 0.014). C_rs_ was significantly higher in *Col5a1*+/− males and females (*p* = 0.007 and *p* = 0.017, respectively) and, as expected, Ers was significantly reduced in *Col5a1*+/− males (*p* = 0.006) and females (*p* = 0.016) (Figure 2). A broadband frequency‐forced oscillation approach (Quick Prime‐3) was used to measure Newtonian airway resistance (R_n_), tissue damping (G), tissue elasticity (H), and tissue hysteresivity (eta; G/H) and there were no statistically significant differences between the *Col5a1*+/− and WT mice (Figure S1).

#### 3.2.2 Partial pressure–volume curves

**FIGURE 3:**
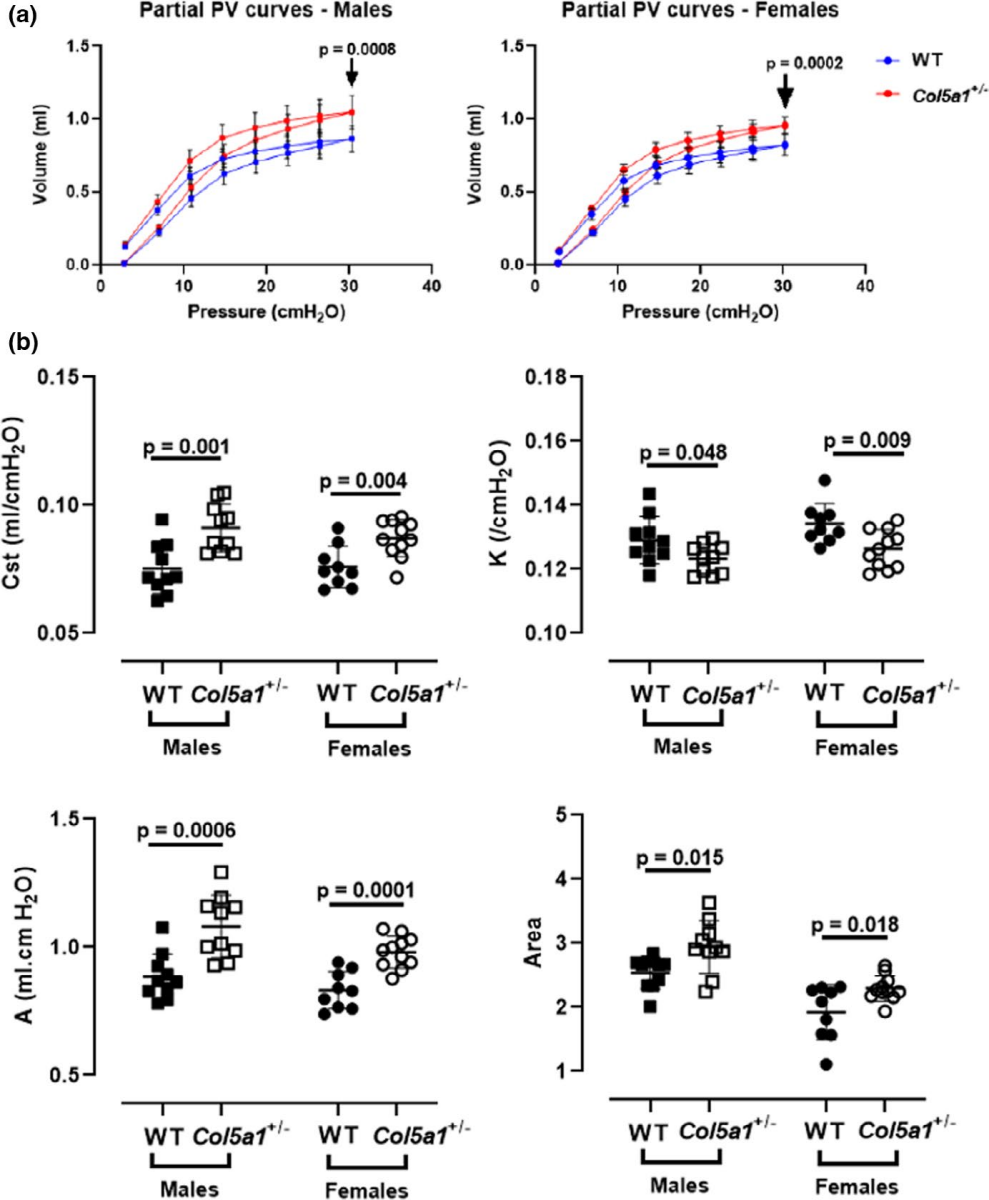
(a) Male and female partial pressure–volume (P‐V) curves. (b) All parameters derived from the P–V curves, including static compliance (C_st_), *k*, A (an estimate of inspiratory capacity), and area were significantly reduced in *Col5a1*+/− compared to control mice (*n* = 10 males, *n* = 9–11 females). Student's *t*‐test

Partial pressure–volume (PV) curves showed a clear upward shift in *Col5a1*+/− mice compared to controls (Figure 3a). At the maximal inspiratory pressure of 30 cm H_2_O, both male and female *Col5a1*+/− mice reached significantly higher volumes compared to WT controls (*p* = 0.0008 and *p* = 0.0002, respectively) (Figure 3a). Quasi‐static compliance (Cst) in *Col5a1*+/− mice was significantly higher when compared to WT in both males and females (*p* = 0.001 and *p* = 0.004, respectively) (Figure 3b). The curvature parameter (K) of the deflation limb of the PV loop was significantly lower in *Col5a1*+/− males and females when compared to WT (*p* = 0.048 and *p* = 0.009, respectively). The derived estimate of inspiratory capacity (A) was markedly elevated in *Col5a1*+/− males (*p* = 0.0006) and females (*p *= 0.0001) when compared to WT. The area enclosed by the inflation and deflation limb of the PV curve was larger in both male and female *Col5a1*+/− mice compared to WT (*p* = 0.015 and *p* = 0.018) (Figure 3b).

#### 3.2.3 Full‐range pressure–volume curves and lung volume measurements

**FIGURE 4:**
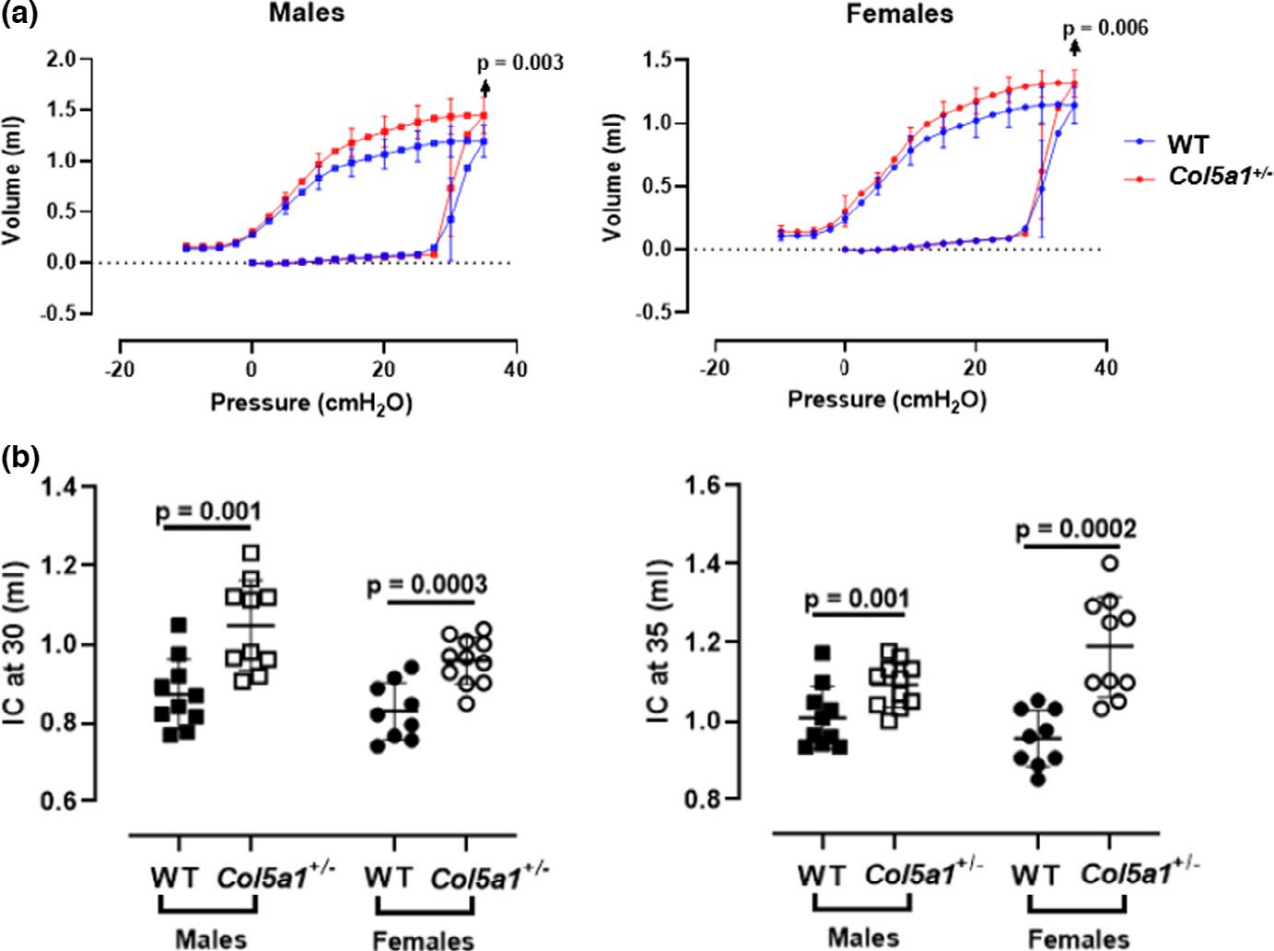
(a) Full‐range pressure–volume curves and (b) Inspiratory capacity measurements derived from deep inflation maneuvers at 30 and 35 cm H_2_O of pressure. (*n* = 10 males, *n* = 9–11 females). Student's *t*‐test

Full‐range PV curves also showed a clear upward shift in male and female *Col5a1*+/− mice compared to wild‐type controls (Figure 4a). In *Col5a1*+/− mice, the inspiratory volume was higher at the maximum pressure (35 cm H_2_O) in male and female mice (*p* = 0.003 and *p* = 0.006, respectively) compared to controls (Figure 4a). Derived values for inspiratory capacity at static pressures were significantly higher at 30 cm H_2_O (*p* = 0.001 and *p* = 0.0003 for males and females, respectively) and 35 cm H_2_O (*p* = 0.001 and *p* = 0.0002 for males and females, respectively) when compared to WT mice (Figure 4b).

**FIGURE 5:**
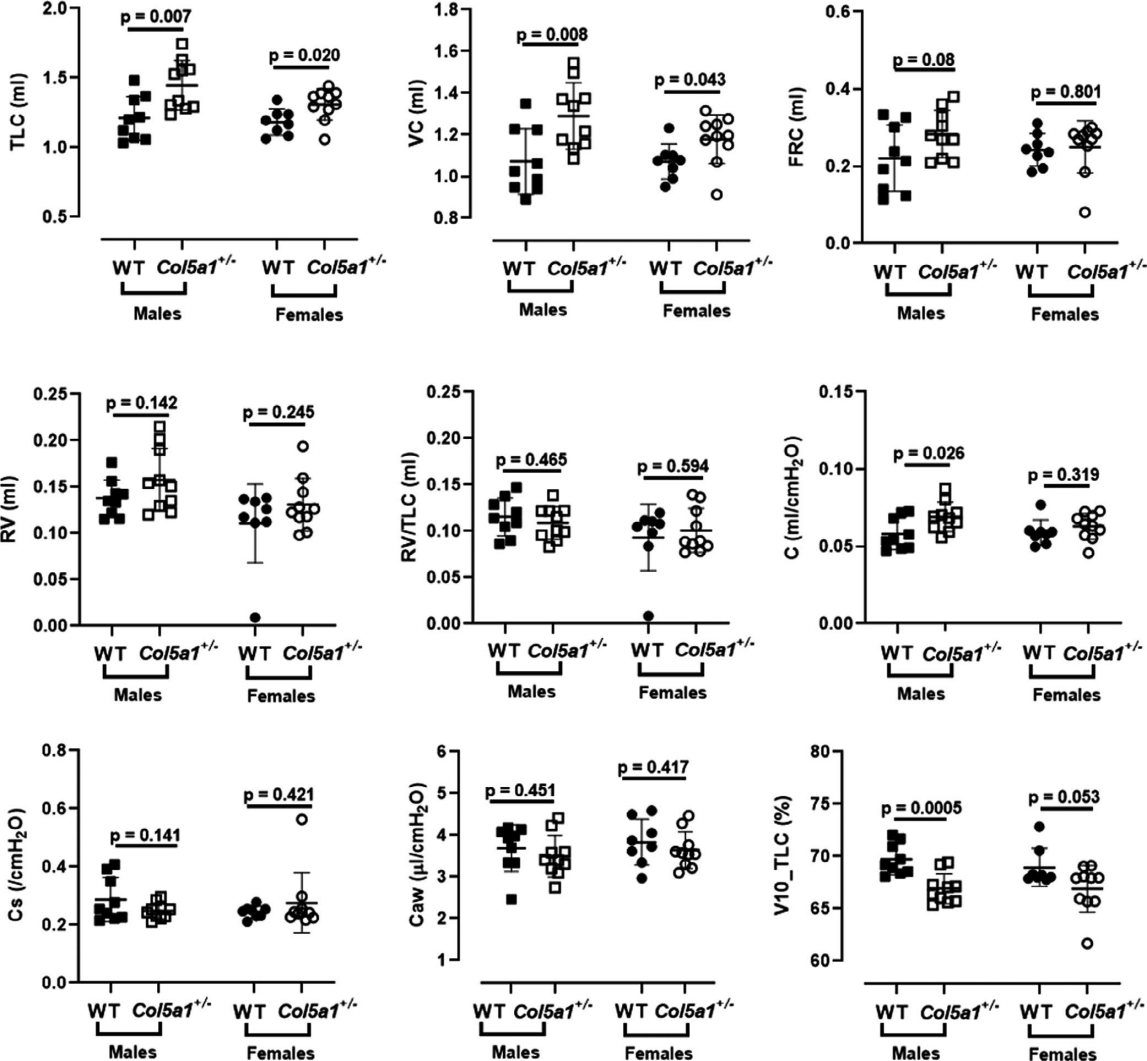
Parameters derived from the lung volume measurements. Total lung capacity (TLC), vital capacity (VC), functional residual capacity (FRC), residual volume (RV), RV/TLC, compliance (C), specific compliance (Cs), airway compliance (Caw), and volume at 10 cm H_2_O as percentage of TLC (V10_TLC). (*n* = 8–10). Student's *t*‐test

Lung volumes and derived variables were calculated from full‐range PV curves. The total lung capacity (TLC) of *Col5a1*+/− was significantly larger compared to WT in males (*p* = 0.007) and females (*p* = 0.02) (Figure 5). Similarly, vital capacity (VC) was significantly elevated in *Col5a1*+/− males (*p* = 0.008), as well as in females (*p* = 0.043), compared to WT controls. The functional residual capacity (FRC) trended to be larger in male *Col5a1*+/− compared to WT (*p* = 0.08) but not in female mice (*p* = 0.801) (Figure 5). Compliance was significantly higher in *Col5a1*+/− male mice compared to WT controls (*p* = 0.026) but not in female *Col5a1*+/− mice (Figure 5). Specific compliance (C_s_) (compliance normalized to FRC) was not different in *Col5a1*+/− males (*p* = 0.141) or females (*p* = 0.421) when compared to WT. Residual volume (RV), RV/TLC, and airway compliance (C_aw_) did not differ in *Col5a1*+/− compared to WT mice. V10_TLC, an index of the shape of the PV curve, was significantly diminished in *Col5a1*+/− males (*p* = 0.0005) with a similar trend in females (*p* = 0.053) (Figure 5).

#### 3.2.4 Negative pressure‐driven forced expiration (NPFE) measurements

**FIGURE 6:**
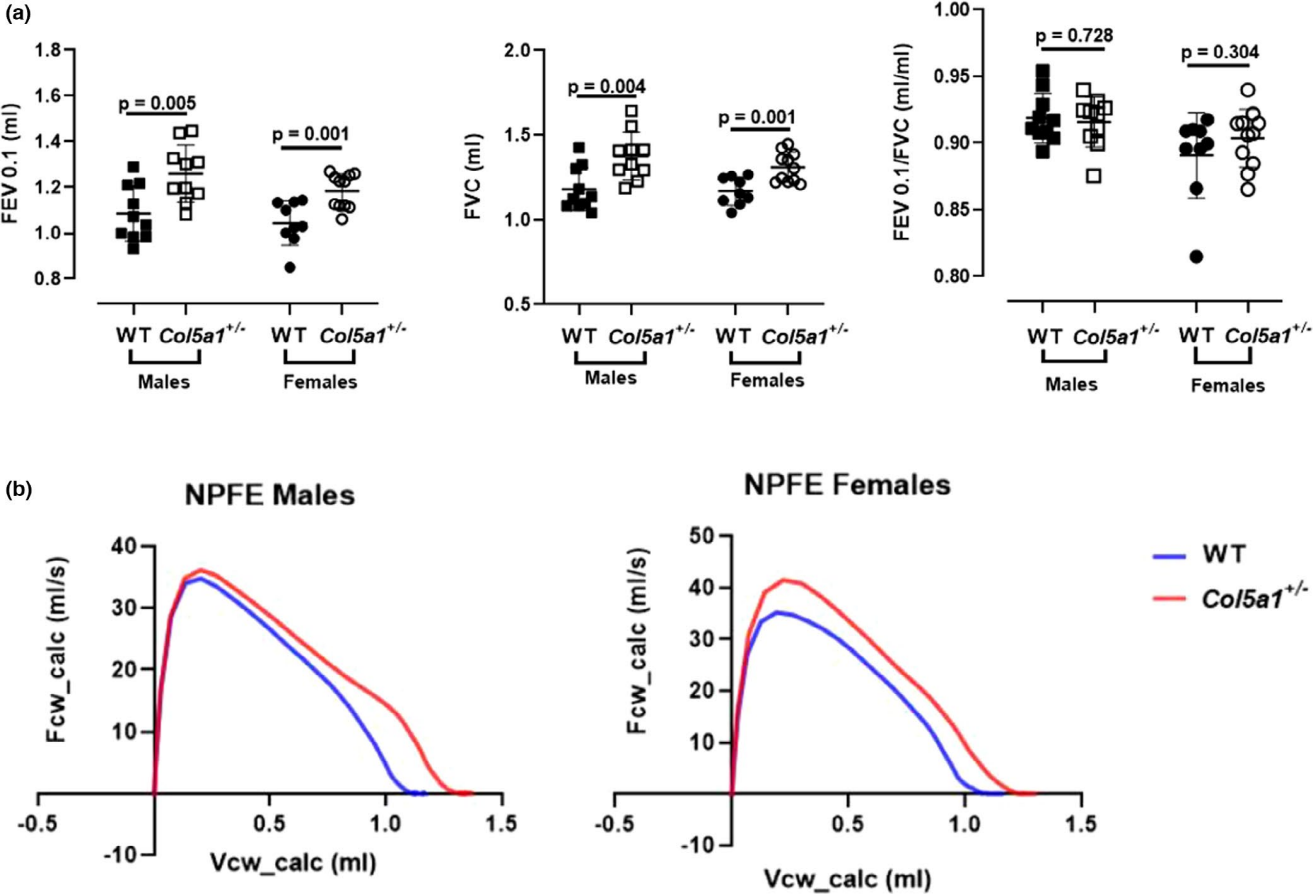
(a) Selected measurements derived from the negative pressure‐driven forced expiration (NPFE) maneuver and (b) flow–volume curves (*n* = 10 males, *n* = 9–11 females). Student's *t*‐test

NPFE maneuvers showed that the forced expiratory volume at 0.1 s (FEV 0.1) was significantly higher in *Col5a1*+/− males and females compared to WT (*p* = 0.005 and *p* = 0.001, respectively) (Figure 6a). Forced vital capacity (FVC) was also higher in *Col5a1*+/− males (*p* = 0.004) and females (*p* = 0.001) compared to controls. There was no difference in the FEV 0.1/FVC ratio in either male or female *Col5a1*+/− mice compared to WT, indicating that the higher FEV 0.1 is proportional to the higher FVC in *Col5a1*+/− mice (Figure 6a). The expiratory limb of the flow volume curves reflected this relationship, with larger volumes throughout the expiratory limb of *Col5a1*+/− mice (Figure 6b). All of the parameters measured by the NPFE maneuver are presented in Table S1.

## 4 DISCUSSION

Although a variety of respiratory manifestations occur in patients with EDS and general observations of lung abnormalities have been reported in a few animal models of EDS (Vroman et al., 2021), comprehensive characterization of respiratory system function has not been previously reported in an EDS mouse model. Using the *Col5a1*+/− mouse model that mimics classical EDS, we found parenchymal lung defects that were accompanied by altered respiratory mechanics compared to wild‐type controls. The lung parenchyma showed alveolar simplification and enlarged air spaces reminiscent of emphysematous‐like changes, while respiratory function studies showed increased respiratory system compliance and increased lung volumes compared to controls, without evidence of expiratory airflow obstruction.

The ECM is important for a healthy lung and alterations in composition, quantity, and post‐translational modification of ECM components underlie important adult respiratory diseases such as idiopathic pulmonary fibrosis (IPF) and chronic obstructive pulmonary disease (COPD) (Burgess et al., 2016). The impact of congenital mutations of collagens on lung development and the respiratory function is less well understood. Type I, III and IV collagens are the most abundant collagens in the lung (Burgstaller et al., 2017; Naba et al., 2012). Recent data, including from our group, demonstrated that type I collagen mutations causing osteogenesis imperfecta (OI), in addition to skeletal manifestations, also generate early morphological lung defects resulting in reduction of alveoli and increased air‐spaces (Baglole et al., 2018; Dimori et al., 2020; Thiele et al., 2012). Changes in respiratory mechanics have also been described in at least one mouse model of OI (Dimori et al., 2020). Heterozygous pathogenic variants in type III collagen cause vEDS, a severe type of EDS characterized by tissue fragility affecting arteries and hollow organs. In vEDS, respiratory complications have been documented and often required clinical intervention (Chohan et al., 2021; Parducci, 2021). This is likely due to the deleterious effects of type III collagen mutations on blood vessels, airways, and lung parenchyma although investigations on the respiratory consequences of vEDS have not been conducted.

Reduced quantity or structural irregularities in type V collagen, resulting from heterozygous pathogenic variants in *COL5A1* or *COL5A2*, cause cEDS, one of the most common EDS types (Wenstrup et al., 2000). Here, we studied the effects of type V collagen haploinsufficiency on the murine lung. Type V collagen, typically expressed as a hetero‐trimer formed by two α1(V) chains and one α2(V) chain, is considered a minor fibrillar collagen and constitutes only about 2–5% of the total collagen tissue content (Malfait et al., 2020). Importantly though, type V collagen forms heterotypic fibrils with type I collagen, and plays a key role in the assembly of these fibrils and the regulation of their diameter (Birk, 2001; Wenstrup, Florer, Brunskill, et al., 2004). As a consequence, haploinsufficiency of *COL5A1* results in defective fibril formation with reduction in fibril count as well as density of those fibrils that are formed (Wenstrup, Florer, Cole, et al., 2004). As such, the early role of type V collagen in the process of heterotypic collagen fibrils nucleation is expected to affect and reduce the deposition of more widespread distributed collagens in the lung, such as type I and III, and manifest in lung tissue changes similar to emphysema. Panacinar emphysema was described in at least one EDS patient in conjunction with reduced, irregular, and frayed in appearance dermal collagen fibrils (Cupo et al., 1981). These defects are likely to be early onset and to impair alveolar formation, similar to what was described in *Adamts2*‐/‐ mice (a mouse model of dermatosparaxis dEDS) (Le Goff et al., 2006) and in mouse models of OI with alterations in type I collagen (Baglole et al., 2018; Baldridge et al., 2010; Dimori et al., 2020; Grafe et al., 2014). Interestingly, however, in cEDS patients respiratory manifestations seem less common than in OI patients. They are not part of the minor clinical criteria for this EDS type (Malfait et al., 2020) nor have been described in a recent cohort of 75 patients with genetically confirmed cEDS (Ritelli et al., 2020). This suggests that intrinsic respiratory and lung changes may exist in cEDS patients but their manifestations are mild or subclinical in otherwise healthy individuals. However, their potential impact on respiratory infections or other respiratory disease processes or insults may be significant.

In order to study the effects of altered collagen and their possible impact on lung function, we performed a comprehensive characterization of respiratory system function utilizing the force oscillation technique, pressure–volume (PV) measurements, and negative pressure‐driven forced exhalation. FOT measurements showed significantly increased Crs and reduced Ers in male and female mice and decreased Rrs in male *Col5a1*+/− mice, consistent with a respiratory system that is more distensible and compliant likely due to disruption in the normal architecture of lung tissue. In female mice, there was a trend toward decreased Rrs that did not meet statistical significance. The reason for this dissimilarity between males and females is unclear. Multiple studies have described sex‐related differences in COPD phenotypes, showing that women are significantly more likely to develop COPD than men (Foreman et al., 2011; Martinez et al., 2007; Pinkerton et al., 2015). Consistent findings, including in non‐smokers with COPD, showed a predisposition to an airway phenotype in females and an emphysema phenotype in males (Hardin et al., 2016; Hong et al., 2016). Sex‐related differences in pulmonary mechanics have also been reported in mouse models with chronic smoke exposure (Tam et al., 2016). Although respiratory mechanics parameters in cEDS mice were consistent with an emphysema‐like pattern in both sexes, some of the measured differences appeared to be of larger magnitude in male vs. female mice, reminiscent of the sex‐related differences observed for COPD. The significance of this is unclear as so little is known about EDS pulmonary phenotypes but represents an interesting finding that merits further studies in the EDS population.

Broadband FOT (Quick Prime‐3) was used to assess possible changes in tissue mechanics in WT vs. *Col5a1*+/− mice. Our results did not reveal statistically significant differences in any of these parameters. Given the emphysema‐like changes observed in *Col5a1*+/− mice histological sections and from histomorphometric measurements, decreased R_n_ and tissue elasticity (H) may be expected, as demonstrated in other mouse models of emphysema (Vanoirbeek et al., 2010). In the present study, tissue elasticity (H) trended lower in *Col5a1*+/− mice, as anticipated, but did not reach statistical significance. This may reflect that the emphysema‐like lung changes in cEDS mice are less severe than in elastase‐induced emphysema, resulting in a milder phenotype. In a model of protease‐induced emphysema in BALB‐C mice, histological evidence of emphysema was present early in the time course when measures of G, H and airways resistance remained similar to controls (Anciaes et al., 2011). Our finding that R_n_ was not different in WT vs. *Col5a1*+/− mice is consistent with our forced expiratory measurements (see below) and, again, may reflect that the emphysema‐like changes are not sufficiently severe in this EDS genotype to affect expiratory airways resistance.

Stepwise partial pressure–volume measurements showed that cEDS mice had greater lung volumes, especially at higher pressures. The relatively higher lung volume on the expiratory limb of the PV curves is greatest between 10 and 30 cm H_2_O, demonstrating the increased respiratory system distensibility of cEDS mice at higher pressures compared to WT. Consistent with this finding, quasi‐static compliance was higher in both males and females. The area enclosed by the partial PV loops is significantly higher in cEDS mice compared to WT, suggesting decreased elastic recoil in the cEDS mice during lung deflation, especially at pressures higher than the tidal breathing range. The K parameter reflects the curvature of the expiratory limb of the PV curve independent of lung volume and represents concavity of the curve toward the pressure axis. In the cEDS mice, K was decreased compared to WT, likely due to the elevation of the PV curve at higher pressures, which leads to less concavity of the PV curve toward the pressure axis and further reflects the relatively lower elastic recoil at higher pressures in cEDS mice. The increased respiratory distensibility in the cEDS mice was especially evident in the “A” parameter of the partial PV curves (Figure 3b), which is an estimate of inspiratory capacity. The full range PV curves clearly replicate the relationship demonstrated in the partial loops, with elevated distention, volume, and flattened slope of the expiratory limb consistent with more easily distensible tissue. These findings are also consistent with the interplay of elastin and collagens in the extracellular matrix (ECM) with elastin bearing the majority of stress at low pressures and volumes and collagen fibers serving as a limit‐step to prevent maximal distention of small airways and alveoli (Toshima et al., 2004).

Lung volume measurements were consistent with elevated FVC measurements and PV loop findings. Total lung capacity (TLC) was higher in cEDS mice when compared to WT, consistent with a respiratory system that is more readily distended to larger volumes. The increase in TLC was due to a greater inspiratory capacity (IC) and vital capacity (VC) and not due to air trapping, as residual volume (RV), FRC and RV/TLC were not significantly elevated. FRC trended higher in male cEDS mice (*p* = 0.08), which likely explains why specific compliance (Cs), compliance normalized to FRC, was not significantly different in cEDS vs. WT mice. V10_TLC, which is the lung volume at 10 cm H_2_O pressure expressed as percent of TLC, was markedly reduced in male and borderline significantly reduced in female cEDS mice compared to WT. This, again, reflects the observation that in *Col5a1*+/− mice respiratory system distensibility was mainly affected at higher pressures. Thus, the volume at 10 cm H_2_O pressure showed only a minor increased while TLC (at 30 cm H_2_O pressure) showed a disproportionately greater increase (Figure 4a). Compliance, as measured during the lung volume protocol, was significantly higher in male cEDS mice compared to WT but not in females. In this protocol, compliance (C) is derived from the slope of the linear portion of the expiratory pressure–volume curve between 3 and 7 cm H_2_O. It is evident from Figure 4a that the slope of this portion of the expiratory PV curve in this pressure range is greater in male cEDS mice compared to WT, while the slopes between 3 and 7 cm H_2_O appear to be very similar for female cEDS mice vs. WT. This pattern is consistent with the observation that the largest differences between cEDS mice and WT are most evident at pressures >10 cm H_2_O. Altered compliance in the male cEDS mice could reflect changes in lung, ribcage, or airway compliance. Airway compliance was not significantly different in cEDS mice vs. WT. Although we suspect that compliance differences are due to ECM alterations in the lungs, we cannot rule out a contribution from the ribcage, which would require open‐chest measurements that are beyond the scope of this study.

Negative pressure‐driven forced exhalation is a maneuver designed to replicate spirometry, demonstrating the relationship between flow and volume during expiration. The results show a significant increase in forced expiratory volume at 0.1 s (FEV0.1), which closely mirrors forced expiratory volume (FEV1) in human subjects, in cEDS mice compared to WT. Forced vital capacity (FVC) was also increased in cEDS mice compared to controls with no difference in the ratio between FEV0.1 and FVC. This indicates that the higher FEV0.1 reflects the larger FVC in cEDS mice, as FEV0.1/FVC was not affected. Interestingly, a study of mostly classical EDS found a high prevalence of dyspnea and FVC, VC, and TLC >120% of predicted in a substantial proportion of subjects, consistent with our results (Morgan et al., 2007). However, the same study also found an elevated RV in >50% of mostly classical EDS patients, which does not agree with our findings. It is possible that this obstructive defect evolves over time and could potentially develop in older mice.

## 5 LIMITATIONS

Although this is the first study to explore respiratory system abnormalities in a mouse model of cEDS, we cannot generalize the findings to other EDS types and it will be important to characterize the lung phenotype of other mouse models mimicking other forms of the disease. For instance, the vEDS type due to mutations in type III collagen appears to be the form of EDS that is most susceptible to pulmonary complications requiring clinical intervention and the study of mice with type III collagen mutations may provide important insights into their specific effects onto the respiratory system. Mutations in *TNXB* cause classical‐like EDS and it has been shown that tenascin‐XB is highly expressed during secondary alveolar septae formation (Foster et al., 2006). Therefore, the study of the lung phenotype in tenascin‐XB mutant mice may provide further insights into the pathology of EDS as it affects the respiratory system. Importantly, potential differences in how collagen mutations impact the respiratory function may exist between rodents and humans and therefore data obtained from mouse models will need to be confirmed in patients.

Another limitation of our study is that all the respiratory measurements were performed in closed‐chest configuration. As such, the potential effects of *Col5a1* haploinsufficiency on the thoracic cage expansion, rib elevation, and diaphragm movement may contribute to the observed pulmonary phenotype. Assessment of their contribution to the observed phenotype would require open‐chest measurements which are beyond the scope of the present study. Lastly, we have performed lung histology and histomorphometry in paraffin‐embedded sections and this process may lead to tissue shrinkage effects which may be genotype specific. Although we have processed samples of both genotypes identically, we cannot exclude potential effects of this issue onto our morphometric measurements.

## 6 CONCLUSION

We have performed the first in‐depth study of the respiratory function in a mouse model of classical EDS and identified significant morphological and functional changes consistent with a more distensible respiratory system, especially at higher pressures. These observations will need to be confirmed in human studies of cEDS patients and may warrant new clinical recommendations and guidelines for the care of these patients.

## AUTHOR CONTRIBUTIONS

JF, MD and RM performed the experiments. JLC and RM conceived the project. All authors contributed to the interpretation of the data and the manuscript preparation. RM is responsible for the integrity of the data.

## Supporting information



Supplementary Material
