# Supplementary material for: Haploinsufficiency of Col5a1 causes intrinsic lung and respiratory changes in a mouse model of classical Ehlers‐Danlos syndrome
Source: Physiol Rep. 2022 Apr 19;10(8):e15275. doi: 10.14814/phy2.15275 (PMC9017971; doi:10.14814/phy2.15275)
Supplement: Supplementary file 1 — Supplementary Material [file PHY2-10-e15275-s001.docx]

| **NPFE** | **WT Males** | ***Col5a1^+/-^* Males** | **WT Females** | ***Col5a1^+/-^* Females** |
| --- | --- | --- | --- | --- |
| FEV 0.1 | 1.085 | **1.260 **** | 1.043 | **1.183 **** |
| FEV 0.05 | 0.984 | **1.104 **** | 0.961 | **1.073 **** |
| FEV 0.2 | 1.111 | **1.298 **** | 1.075 | **1.221 **** |
| FEV_PEF | 0.183 | 0.197 | 0.209 | 0.238 |
| FVC | 1.180 | **1.376 **** | 1.170 | **1.310 **** |
| FEF 0.1 | 0.683 | 0.871 | 0.778 | 0.902 |
| FEF 0.05 | 5.972 | **9.769 **** | 3.880 | 5.232 |
| FEF 0.2 | 0.102 | **0.174 **** | 0.134 | 0.174 |
| FEF_50% FVC | 23.48 | 23.22 | 25.69 | 27.42 |
| PEF | 34.88 | 36.30 | 35.47 | **41.72 **** |
| TPEF | 0.007 | 0.007 | 0.008 | 0.008 |
| FEV0.1/FVC | 0.918 | 0.915 | 0.890 | 0.903 |

**Supplementary Table 1.** All the measurements derived from the negative pressure-driven forced expiration (NPFE) maneuver (**p≤0.01). Student’s *t*-Test. FEV= forced expiratory volume at 0.1 seconds (or 0.05 sec or 0.2 sec); FEV_PEF = forced expiratory volume @ peak expiratory flow; FVC = forced vital capacity; FEF = forced expiratory flow at 0.1 seconds (or 0.05 sec or 0.2 sec); FEF_50% FVC = forced expiratory flow at 50% of forced vital capacity; TPEF = time to reach peak expiratory flow.


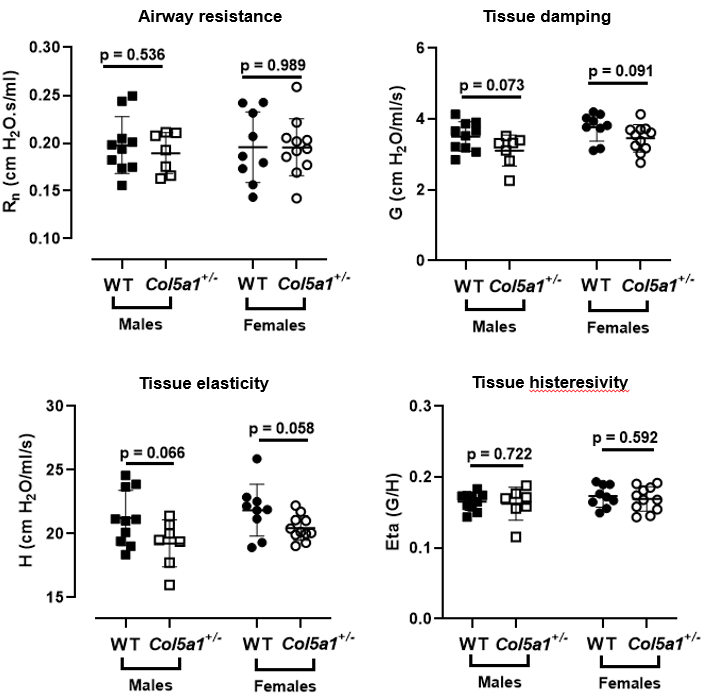


**Supplementary Figure 1.** Measurements of the Newtonian airway resistance (R_n_), tissue damping (G), tissue elasticity (H) and tissue hysteresivity (Eta = G/H) derived from the Quick prime-3 maneuver using the forced oscillation technique. (n = 7-10 males, n = 9-11 females). Student’s *t*-Test.
